# Acetylsalicylic acid and dihydroartemisinin combined therapy on experimental malaria-associated acute lung injury: analysis of lung function and the inflammatory process

**DOI:** 10.1186/s12936-024-05017-7

**Published:** 2024-09-19

**Authors:** Helena D’Anunciação de Oliveira, Camila Nunes Batista, Maiara Nascimento Lima, Ana Carolina Lima, Beatriz Amanda Barbosa Rangel dos Passos, Rodrigo Jose Rocha Xavier Freitas, Johnatas Dutra Silva, Debora Gonçalves Xisto, Marcos Vinícius Rangel-Ferreira, Marcelo Pelajo, Patricia Rieken Macedo Rocco, Flávia Lima Ribeiro-Gomes, Hugo Caire de Castro Faria-Neto, Tatiana Maron-Gutierrez

**Affiliations:** 1 Laboratory of Immunopharmacology, Oswaldo Cruz Institute, Fiocruz, Av. Brasil, 4036 - Bloco 2. Manguinhos, Rio de Janeiro, RJ 21040-361 Brazil; 2 Laboratory of Pulmonary Investigation, Carlos Chagas Filho Institute of Biophysics, Federal University of Rio de Janeiro, Rio de Janeiro, Brazil; 3 Laboratory of Malaria Research, Oswaldo Cruz Institute, Fiocruz, Rio de Janeiro, Brazil; 4 Laboratory of Pathology, Oswaldo Cruz Institute, Fiocruz, Rio de Janeiro, Brazil

**Keywords:** Malaria, Acute respiratory distress syndrome, Lung edema, Dihydroartemisinin, Acetylsalicylic acid

## Abstract

**Background:**

Severe malaria can cause respiratory symptoms, which may lead to malaria-acute lung injury (MA-ALI) due to inflammation and damage to the blood-gas barrier. Patients with severe malaria also often present thrombocytopenia, and the use of acetylsalicylic acid (ASA), a commonly used non-steroidal anti-inflammatory drug with immunomodulatory and antiplatelet effects, may pose a risk in regions where malaria is endemic. Thus, this study aimed to investigate the systemic impact of ASA and dihydroartemisinin (DHA) on ALI induced in mice by *Plasmodium berghei* NK65 (*Pb*NK65).

**Methods:**

C57BL/6 mice were randomly divided into control (C) and *Pb*NK65 infected groups and were inoculated with uninfected or 10^4^ infected erythrocytes, respectively. Then, the animals were treated with DHA (3 mg/kg) or vehicle (DMSO) at the 8-day post-infection (dpi) for 7 days and with ASA (100 mg/kg, single dose), and analyses were performed at 9 or 15 dpi. Lung mechanics were performed, and lungs were collected for oedema evaluation and histological analyses.

**Results:**

*Pb*NK65 infection led to lung oedema, as well as increased lung static elastance (Est, L), resistive (ΔP1, L) and viscoelastic (ΔP2, L) pressures, percentage of mononuclear cells, inflammatory infiltrate, hemorrhage, alveolar oedema, and alveolar thickening septum at 9 dpi. Mice that received DHA or DHA + ASA had an increase in Est, L, and CD36 expression on inflammatory monocytes and higher protein content on bronchoalveolar fluid (BALF). However, only the DHA-treated group presented a percentage of inflammatory monocytes similar to the control group and a decrease in ΔP1, L and ΔP2, L compared to Pb + DMSO. Also, combined treatment with DHA + ASA led to an impairment in diffuse alveolar damage score and lung function at 9 dpi.

**Conclusions:**

Therapy with ASA maintained lung morpho-functional impairment triggered by *Pb*NK65 infection, leading to a large influx of inflammatory monocytes to the lung tissue. Based on its deleterious effects in experimental MA-ALI, ASA administration or its treatment maintenance might be carefully reconsidered and further investigated in human malaria cases.

**Supplementary Information:**

The online version contains supplementary material available at 10.1186/s12936-024-05017-7.

## Background

*Plasmodium falciparum* infection can lead to pulmonary complications in 5–25% of cases, ranging from mild respiratory symptoms to a severe condition known as malaria-associated acute lung injury (MA-ALI) [1]. Although severe malaria is less prevalent in infections caused by *Plasmodium vivax*, common in the Amazonian region, 21.5% of patients presented pulmonary oedema [2]. Moreover, *P. vivax* MA-ARDS fatal cases have been described [3, 4]. Even after anti-malarial treatment, clinical manifestations associated with MA-ALI may persist, and, unfortunately, 50–80% of cases result in fatalities [1, 5]. MA-ALI pathophysiology is complex and involves an exacerbated inflammatory response that disrupts the integrity of the alveolar-capillary membrane. This leads to interstitial and alveolar oedema, leukocyte infiltration, and lung haemozoin accumulation [6–8]. Patients with MA-ALI also present adhesion of *Plasmodium*-infected red blood cells (iRBCs) to capillaries and venules, which can trigger pulmonary capillary obstruction and lung oedema. These events are accompanied by disruption of the blood-gas barrier, leading to massive monocyte infiltration and tissue remodelling [9]. Despite the high mortality rate, no specific MA-ALI treatment is available.

Acetylsalicylic acid (ASA), a widely used non-steroidal anti-inflammatory drug, is often prescribed in low doses to patients with cardiovascular diseases based on recommendations from the World Health Organization (WHO) and the European Society of Cardiology (ESC) [10, 11] that may be used indiscriminately.

Although the use of aspirin in MA-ALI was not previously described, its effects in other aetiologies of acute respiratory distress syndrome (ARDS) have been extensively investigated. Both low or high-dose aspirin led to decreased TNF BALF levels and neutrophilic lung inflammation and pulmonary thromboxane B2 (TXB_2_) in human lung inflammation triggered by LPS [12]. Also, patients at risk of developing ARDS have presented a decrease in plasma levels of TXB2 and the ratio of aspirin-triggered lipoxinA4 (15-epi-LXA_4_)/TXB_2_ [13]. However, a phase 2b trial found that aspirin administration did not significantly reduce the incidence of ARDS at 7 days [14]. Regarding experimental models, previous studies revealed opposite mechanisms associated with ASA administration in cerebral malaria (CM) models. Studies by McMorran and colleagues have shown that ASA’s irreversible binding on cyclooxygenase-1 (COX-1) serine-529 terminal leads to platelet inhibition, reducing infected erythrocytes clearance and worsening CM pathogenesis [15]. However, platelet inhibition reduces vascular obstruction in the CM model [16].

Herein, other independent COX-1 effects of ASA associated with its anti-inflammatory properties were investigated. Although ASA can modulate and mitigate cell rolling and diapedesis, it also induces CD36 expression in human monocytes [17]. CD36 has been shown in the *Plasmodium berghei* strain ANKA (*Pb*ANKA) model as a molecule involved in infected-erythrocyte phagocytosis mediated by monocyte-derived macrophages, leading to higher lung tissue damage [5, 18]. Therefore, the effects of ASA treatment in the context of malaria must be evaluated. While ASA has benefits in specific scenarios, its impact on malaria should be considered in the context of the disease’s complex pathophysiology.

Although chloroquine is still effective against *P. vivax* infection in Brazil, the WHO guidelines indicate artemisinin-based combination therapy (ACT) for severe malaria and treating *P. falciparum* infection. Dihydroartemisinin (DHA)—a semisynthetic derivative of artemisinin and active artesunate metabolite [19]—was an anti-malarial treatment. Thus, the effects of using ASA combined with DHA on survival rate, morpho-functional parameters, and monocyte recruitment in experimental MA-ALI were aimed to be evaluated.

## Methods

### Experimental protocol

A total of 180 male C57BL/6 mice (18–20 g, 8–12 weeks old) were randomly divided into control (C) and *P. berghei* strain NK65 (*Pb*NK65) infected groups and were inoculated with uninfected or 10^4^ infected erythrocytes, respectively. The animals were treated at the 8 dpi with acetylsalicylic acid (ASA) (100 mg/kg) or saline orally and daily, from the 8 to the 14 dpi with dimethyl sulfoxide (DMSO) or DHA (3 mg/kg), intraperitoneally (i.p.). DHA vehicle was determined in a set of independent experiments using chloroquine (CQ) (25 mg/kg) orally, or DHA (3 mg/kg) solubilized in saline, DMSO or 60:40 DMSO: Polysorbate 80 (PS), intraperitoneally (Fig. S1). The percentage of infected erythrocytes and blood leukocytes in the peripheral blood was determined by microscopic analysis using the panoptic method (Laborclin, PR, Brazil).

### Lung mechanics

From the 6 to the 10 dpi after infection, animals were sedated (diazepam, 1 mg, i.p.), anesthetized (thiopental sodium, 20 mg/kg, i.p.), tracheotomized, paralyzed (vecuronium bromide, 0.005 mg/kg, intravenously, i.v.), and mechanically ventilated with a constant flow ventilator (Samay VR15; Universidad de la Republica, Montevideo, Uruguay) using the following parameters: tidal volume, 0.2 mL; respiratory rate, 100 breaths/min; and the fraction of inspired oxygen, 0.21. The anterior chest wall was surgically removed, and a positive end-expiratory pressure of 2 cm H_2_O was applied. In an open-chest preparation, tracheal pressure reflects transpulmonary pressure [20]. Static lung elastance (Est, L), the pressure spent to overcome airway resistance (ΔP1, L), and stress relaxation or viscoelastic properties of the lung (ΔP2, L) were measured using ANADAT data analysis software (RHT-InfoData, Inc., Montreal, Quebec, Canada). Lung mechanics were also performed from 7 to 10 dpi.

### Index of lung oedema

As previously described, the lung wet-to-dry weight ratio was used as an index of pulmonary oedema formation [21]. Briefly, the lungs were isolated, and large airways were removed. Both lungs were weighed and then dried in a microwave at low power (200 W) for 30 s. The drying process was repeated until the difference between the two consecutive lung weight measurements was less than 5%. The last weight measurement represented the dry weight.

### Histological analysis

After determining lung mechanics, laparotomy was performed, and heparin (1000 IU i.v.) was injected into the left ventricle. The trachea was clamped at the end-expiration, and the abdominal aorta and vena cava were sectioned. The left lung and liver were then isolated, fixed with formaldehyde 10%, and embedded in paraffin. Slices were cut (4-μm thick), deparaffinized, and stained with haematoxylin and eosin. Diffuse alveolar damage (DAD) was quantified using a weighted scoring system by a researcher blinded to the experimental protocol. Briefly, scores from 1 to 5 were used to represent alveolar oedema, septal thickening, haemorrhage, inflammatory infiltration, and microatelectasis, with 1 standing for no effect and 5 for maximum severity. In addition, the extent of each scored characteristic per field of view was determined on a scale of 1 to 5, with 1 standing for no visible evidence and 5 for complete involvement. Scores were calculated as the product of severity and the extent of each feature and thus ranged from 0 to 25. One pathologist expert in lung histology assessed scoring. The assessor was blinded by the group assignment.

The percentage of mononuclear and polymorphonuclear cells in the lung tissue were analysed in 4-μm thick sections stained with haematoxylin–eosin, by using the point-counting technique. Briefly, an integrating eyepiece with a coherent system consisting of a grid with 100 points and 50 lines of known length was coupled to a conventional light microscope. Then, total leukocytes and differential cell count were performed across ten random, non-coincident microscopic fields at a magnification of 1000×, per mouse (Olympus BX51, Olympus Latin America-Inc., Brazil) [22].

### Bronchoalveolar lavage fluid analysis

The bronchoalveolar lavage fluid (BALF) was carried out via a tracheal tube with phosphate-buffered saline solution (1 ml). Bronchoalveolar lavage fluid was centrifuged at 4 °C for 10 min at 400×*g*, the cell pellet was resuspended in phosphate-buffered saline (PBS) for leukocyte enumeration, and the supernatant was removed and stored at − 80 °C for further analyses. Total leukocyte numbers were measured in a Neubauer chamber under light microscopy after diluting the samples in Türk solution (2% acetic acid). Differential cell counts were performed in cytospin smears stained by the panoptic method (Laborclin, PR, Brazil). BALF total protein concentration was measured using a BCA protein assay kit (Thermo Scientific, Waltham, MA, USA). The protein profile was analysed by reducing sodium dodecyl sulfate–polyacrylamide gel electrophoresis (12% acrylamide).

### Blood count and biochemical analysis

Blood samples were collected by cardiac puncture, transferred to collection tubes for blood count and biochemistry, and sent for analysis in the Laboratory Animal Clinical Analysis Platform at Fiocruz. Blood count analyses were performed on the Poch 100 iV-DIFF automated hematology analyzer (Sysmex, Kobe, Japan). Hepatic and renal functions were also automated and analysed using the Vitros 250 (Ortho clinical—Johnson & Johnson, New Brunswick, New Jersey, USA).

### Lung processing

Mice were anesthetized and, after bronchoalveolar lavage, perfused with 20 ml of PBS. The lungs were individually harvested, minced with scissors, and incubated in 500 μl RPMI containing 100 U/ml penicillin, 100 μg/ml streptomycin, and 128 μg/ml collagenase type I for 1 h at 37 °C. After this time, additional 4500 μl of RPMI with 5% SFB and 0.05% DNAse were added, and the tissue was passed through a 70 μm nylon cell filter to complete dissociation and separate the individual cells. The resulting single-cell suspensions were washed and labeled for flow cytometry analysis.

### Phenotypic analysis by flow cytometry

Lung single cells were stained with Live/Dead Fixable Violet (Invitrogen) according to the manufacturer’s recommendations to exclude dead cells. They were then washed and incubated for 20 min on ice with the following antibodies: anti-Fc-γ III/II (CD16/32) receptor (2.4G2; BD Pharmingen), FITC anti-mouse CD45 (30-F11; BD, Pharmingen), Alexa Fluor 700 anti-mouse CD11b (M1/70; BD, Pharmingen), APC anti-mouse Ly6G (1A8; BD, Pharmingen), APC Cy7 anti-mouse Ly6C (AL-21; BD, Pharmingen), PE- anti-mouse CD36 (HM36; eBioscience). Data was collected with a CytoFLEX Flow Cytometer (Beckman Coulter) and analysed with FlowJo software (BD Bioscience). At least 1 million cells were acquired per sample.

### Enzyme-linked immunosorbent assay

Levels and monocyte chemoattractant protein (MCP-1) were quantified in the BALF by ELISA according to the manufacturer’s instructions (R&D Systems, Minneapolis, MN, USA).

### Statistical analysis

The normality of the data was tested using the Kolmogorov–Smirnov test with Lilliefors’ correction. The Levene median test was used to evaluate the homogeneity of variances. If both conditions were satisfied, one-way ANOVA followed by Tukey’s post hoc test was used. Parametric data were expressed as means ± standard error (SEM). For nonparametric results, the Kruskal–Wallis test, followed by Dunn’s test, was used. Nonparametric data were expressed as boxplots showing the interquartile (P25–P75) range, with whiskers denoting the range (minimum–maximum) and horizontal lines representing the median. All tests were performed using the Prism 9.0 software package (GraphPad Software Inc., La Jolla, CA, USA). In all tests, the significance level was set at 5%.

## Results

### Morpho-functional lung dysfunction in *Plasmodium berghei* NK65 infected mice

**Fig. 1:**
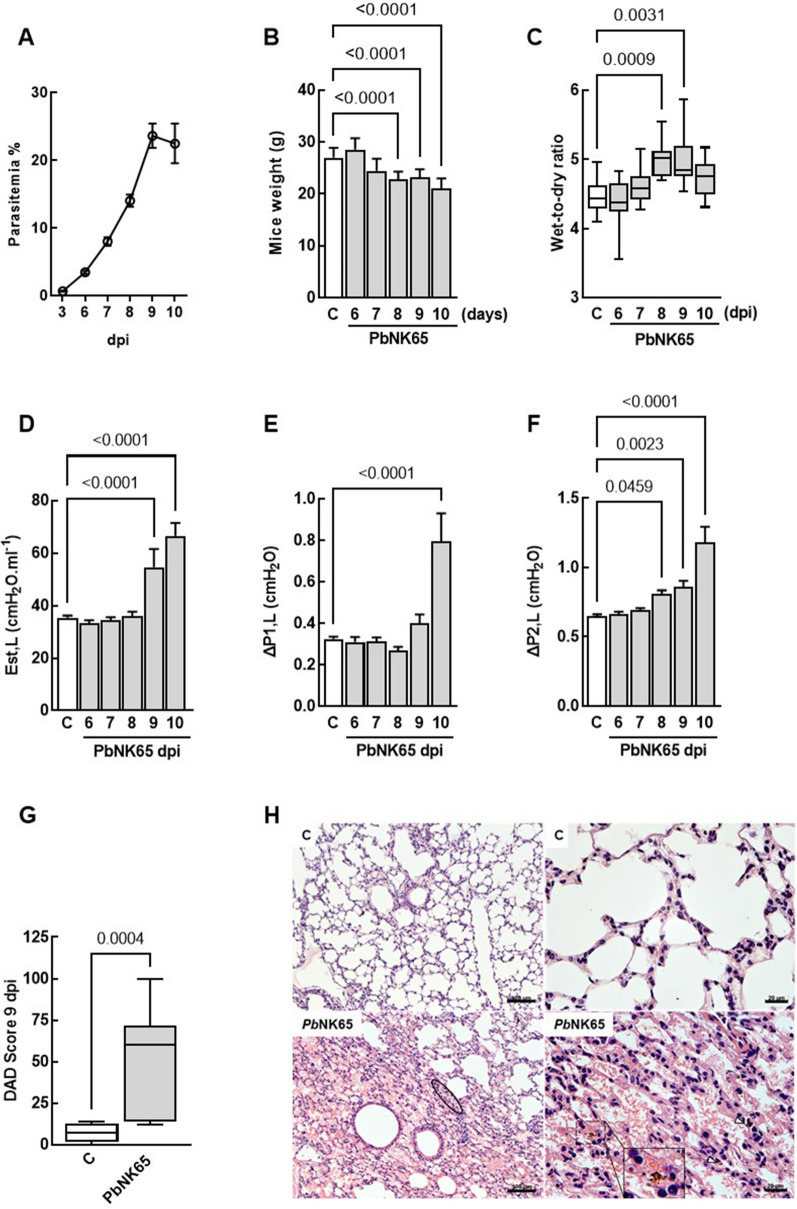
*Pb*NK65 infection led to lung oedema and impairment of lung mechanics. Mice were infected with 10^4^ *Pb*NK65 parasitized RBCs or uninfected RBCs. Peripheral parasitaemia (**A**), mice weight (**B**), lung oedema (**C**), lung static elastance (Est, L) (**D**) Resistive (ΔP1, L) (**E**) and viscoelastic (ΔP2, L) (**F**) pressures were assessed from 6 to 10 dpi. A semiquantitative, severity-based score (1–5) was used to measure diffuse alveolar damage (DAD) (**G**). Representative photomicrographs of lung parenchyma stained with hematoxylin and eosin (**H**). Note that the *Pb*NK65-infected group lung damage was observed, with areas of alveolar collapse (circle, lower left panel), alveolar septal inflammation and presence of haemozoin (inlet and white arrowhead, lower right panel). Data are represented as means ± SEM, and DAD score are given as medians (interquartile ranges) of 7–10 animals in each group. Bars: 100 μm (left panels, and 20 μm (right panels)

*Pb*NK65 induced MA-ALI with increased parasitaemia (Fig. 1A) and loss of weight from 8 to 10 dpi (Fig. 1B), as previously described [23]. Although the *Pb*NK65 model is well established, lung function analysis was still poorly analysed. Thus, the presence of pulmonary oedema and the increase of static lung elastance (Est, L), airway resistance (ΔP1, L), and stress relaxation pressures (ΔP2, L) were investigated from 6 to 10 dpi. *Pb*NK65 infection significantly increased lung oedema compared to control at 8 and 9 dpi (Fig. 1C). Also, Est, L was higher in the *Pb*NK65 group at 9 and 10 dpi compared to the C group (Fig. 1D), while ΔP1, L and ΔP2, L were increased at 10 dpi and from the 8 to 10 dpi, respectively (Fig. 1E, F). Diffuse alveolar damage (DAD) score analysis confirmed that *Pb*NK65 infection led to significant histological alterations at 9 dpi (Fig. 1G). At 9 dpi, photomicrographs of lung tissue samples collected from the control and *Pb*NK65-infected groups showed a notable presence of inflammatory cells, particularly mononuclear cells, in the lung parenchyma and alveolar spaces. In addition, fluid leakage into the alveolar space and thickening of the alveolar septum, oedema, and microatelectasis areas in the infected animals (Fig. 1H). Together, these results suggest that the onset of lung injury occurs at 8 dpi in *Pb*NK65-infected mice.

### Effect of solubilization agents on the efficacy of dihydroartemisinin treatment in *Plasmodium berghei* NK65-infected mice

**Fig. 2:**
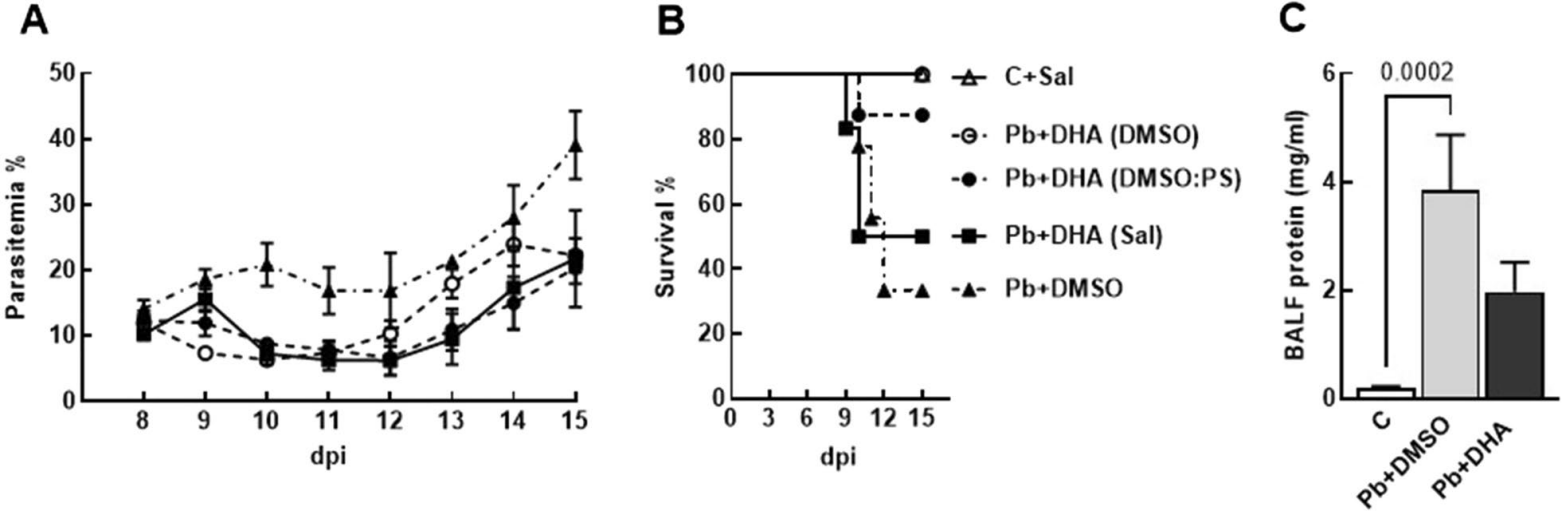
DHA treatment efficacy in *Pb*NK65 infection is dependent on its solubilization. Survival rate (**A**) and parasitaemia (**B**) were monitored daily after inoculation with *Pb*NK65. Mice were treated from 8 to 14 dpi with DHA solubilized in saline (SAL), dimethyl sulfoxide (DMSO), or 60:40 DMSO: Polysorbate 80 (DMSO: PS), intraperitoneally. BALF samples from control, infected, and treated animals with DHA were analysed at 9 dpi. The protein concentration was determined by BCA (**C**). Data are represented as means ± SEM of 7–11 animals in each group

Given that DHA is among the anti-malarial drugs recommended for severe malaria treatment and that previous research lacks a standardized protocol for its administration [24–26], an evaluation of various solubilization agents for DHA was conducted. Peripheral parasitaemia was reduced 24 h after CQ or DHA solubilized in DMSO and DMSO: PS and in all treated groups compared to *P. berghei* + DMSO at 10 dpi. All DHA-treated groups had a similar progression of parasitaemia from 12 to 15 dpi (Fig. 2A). The DHA + DMSO group presented a 100% survival rate (p < 0.004) compared to the untreated Pb + DMSO group. When solubilized in DMSO: PS, 87.5% of DHA-treated groups survived until 15 dpi (p < 0.038) compared to the untreated (Pb + DMSO) group. However, only 50% of animals treated with DHA solubilized in saline survived, which was not significantly different from the untreated group (33.3%) (Fig. 2B). Thus, DMSO was considered the most suitable vehicle for DHA treatment.

Considering the differences between DHA-treated groups on the survival rate, it was investigated whether DHA treatment modulated the blood-gas barrier permeability. *Pb*NK65 infection, but not the DHA-treated group, led to an increase in protein content on BALF (Fig. 2C) at 9 dpi, compared to the C group. These data indicated that DHA solubilized in DMSO might mitigate parasitaemia levels as well as the protein content on BALF, with an increased survival rate in the *Pb*NK65 MA-ALI model.

### DHA and ASA treatment maintained lung tissue damage on *Pb*NK65-infected mice

**Fig. 3:**
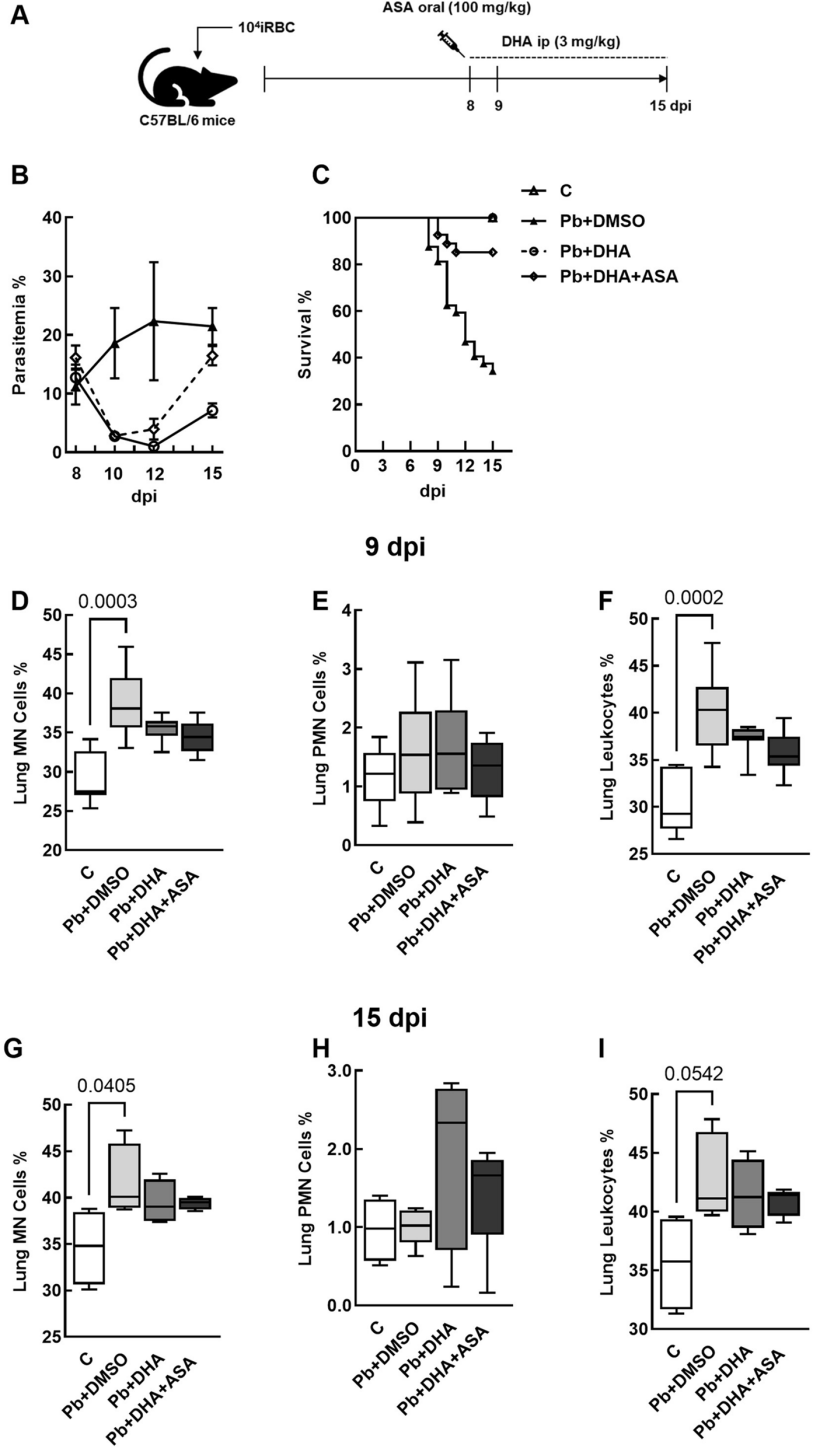
Effects of DHA and ASA on survival and differential cellularity in the lung. Experimental design (**A**). Survival rate (**B**) and parasitaemia (**C**) were monitored daily. Mononuclear cells, polymorphonuclear cells, and total leukocytes were quantified in the lung tissue at 9 (**D**–**F**) and 10 dpi (**G**–**I**) in 10 non-coincident fields of slides stained with HE under magnification of 1000x. Data are represented as means ± SEMTotal and differential cellularity are given as medians (interquartile ranges). N = 7–11 (**B**, **C**), 9–11 (**D**–**F**) and 4–5 (**G**–**I**) animals in each group

Besides COX-1-dependent effects, ASA can modulate leukocyte recruitment, acting on rolling and diapedesis [27]. However, the conflicting data regarding its impact on CM [15, 16] prompted us to evaluate whether treating ASA alone or combined with DHA could influence the survival rate, parasitaemia, lung function, and leukocyte recruitment to the lung tissue. Considering that ASA administration is associated with gastric mucosa injury [28], the treatment with a single dose of ASA at 8 dpi was chosen, and DHA was administered i.p. from 8 to 14 dpi (Fig. 3A). DHA treatment led to a 100% survival at 15 dpi, while only 34.4% of *Pb*NK65 animals survived. The combined therapy of DHA + ASA resulted in a survival rate of 85.2% (Fig. 3C). DHA alone or combined with ASA reduced parasitaemia at 10 and 12 dpi but not at 15 dpi (Fig. 3B).

The increase in protein content on BALF (Fig. 2C) prompted us to determine whether DHA or ASA treatment would also modulate leukocyte recruitment to the lung tissue. Differential cellularity in the lung revealed that *P. berghei* + DMSO group presented more mononuclear cells (Fig. 3D) and total leukocytes (Fig. 3F) on lung tissue compared to control mice at 9 dpi. However, no differences in polymorphonuclear cell count were observed (Fig. 3E). At 15 dpi, polymorphonuclear cells (Fig. 3H) count remained similar between control and infected groups, whereas the increase in mononuclear population was maintained in the untreated group (Fig. 3G). These data suggest that DHA alone or combined with ASA promotes a transient influx of mononuclear cells to lung tissue in experimental MA-ALI.

Comparable to human infections, *Pb*NK65-infected animals exhibited liver histological changes, such as leukocyte interaction with infected and non-infected red blood cells in the central vein lumen. Furthermore, hyperplasia of Kupffer cells containing phagocytosed haemozoin was observed in all infected animals, being more subtle in the Pb + DHA group (Fig. S1A). *Pb*NK65 infection decreased serum albumin and alkaline phosphatase levels and increased aspartate aminotransferase (AST) levels compared to the control group. In addition, only *P. berghei* + DMSO group showed an increase in alanine aminotransferase (ALT) levels (Fig. S1B–E).

Severe malaria is commonly characterized by an increase in the blood–brain barrier (BBB) permeability and severe anaemia. This feature can be reproduced in experimental models, using *Pb*ANKA strain. Herein, Evans’s blue leakage assay showed that both control and all *Pb*NK65-infected groups had no significant dye accumulation, indicating that BBB remained intact (Fig. S2A, B). However, *Pb*NK65 infection led to anaemia and thrombocytopenia (Fig. S2C–F). Sodium, urea, and creatinine serum levels were measured, and no differences between control and infected groups were observed, suggesting that *Pb*NK65 does not lead to kidney injury.

### Exacerbation of lung morpho-function impairment in the *Pb*NK65 model by ASA: evidence from histopathological, functional, and immunological evaluations

**Fig. 4:**
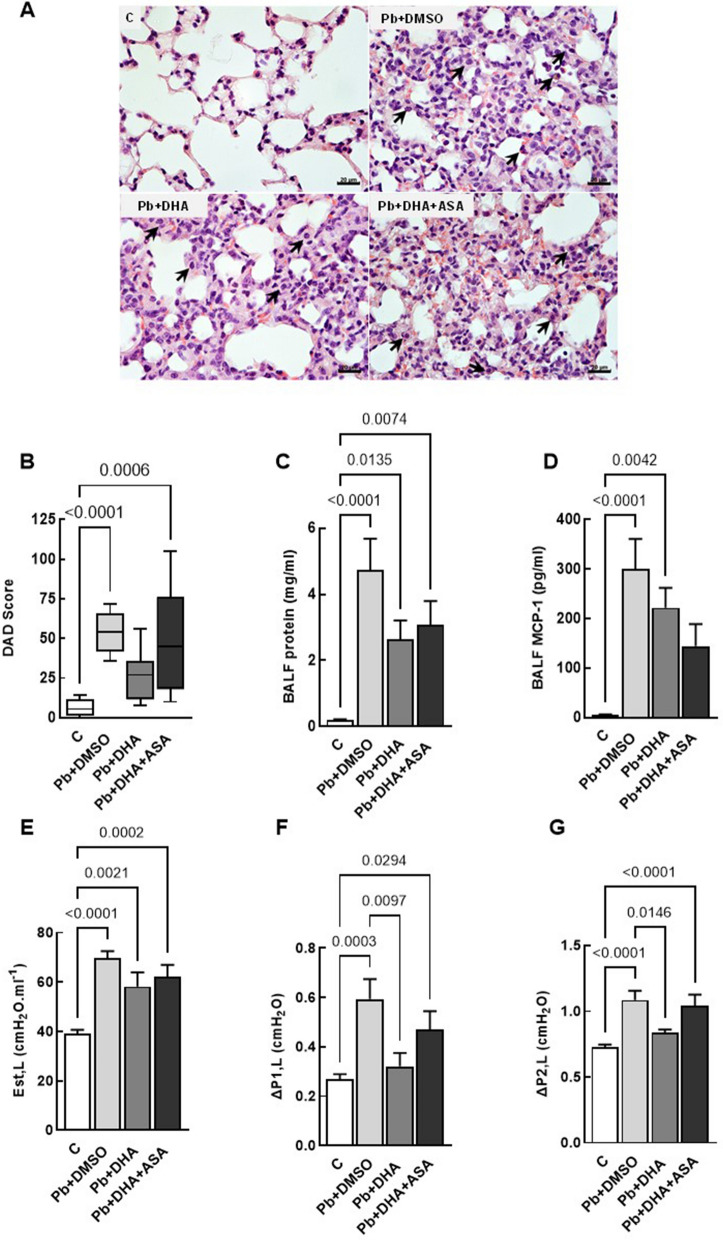
Lung morpho-functional impairment in experimental MA-ALI was maintained 24 h after DHA or DHA + ASA treatment. Representative photomicrographs of lung parenchyma stained with hematoxylin and eosin (**A**). DAD score (**B**), protein content on BALF (**C**) lung static elastance (Est, L) (**E**), resistive (ΔP1, L) (**F**) and viscoelastic (ΔP2, L) pressures (**G**) were measured at 9 dpi. MCP-1 levels were assessed by ELISA (**D**). Data are represented as means ± SEM, and DAD score are given as medians (interquartile ranges) of 10–21 animals in each group. Bars: 100 μm

In contrast to other causes of ALI, *Plasmodium* spp. human infections are characterized by a significant migration of macrophages rather than neutrophilic infiltration [29]. In this study, the *P. berghei* + DMSO, *P. berghei* + DHA and *P. berghei* + DHA + ASA groups exhibited inflammatory infiltrations in the lung tissue. The animals treated with DHA + ASA showed alveolar septal thickening, alveolar oedema, and haemozoin phagocytosis by alveolar macrophages, while these parameters were less severe in animals that received only anti-malarial treatment. The DMSO and DHA + ASA groups also had a higher diffuse alveolar damage (DAD) score than the control group, while animals that received only anti-malarial treatment did not present significant histological alterations (Fig. 4A, B).

Subsequently, it was evaluated whether ASA could increase blood-gas barrier permeability by measuring the protein content on the BALF. The *P. berghei* + DMSO group presented a remarkable increase in protein levels on BALF, as also observed in both DHA and DHA + ASA-treated groups. Interestingly, *P. berghei* + DMSO and *P. berghei* + DHA groups presented higher MCP-1 levels compared to the uninfected control group (Fig. 4C, D). This outcome is consistent with the differential cellularity count in the lung tissue, which revealed a preponderance of mononuclear cells after *Pb*NK65 infection.

Therefore, it was investigated whether the tissue damage observed was related to lung function impairment. DHA and ASA treatments increased Est, L, but only the ASA group had higher ΔP1, L and ΔP2, L. Animals that received only DHA treatment had a decrease in ΔP1, L and ΔP2, L compared to the *P. berghei* + DMSO group (Fig. 4C–E). These results suggest that ASA may exacerbate lung morpho-function impairment in the *Pb*NK65 model.

**Fig. 5:**
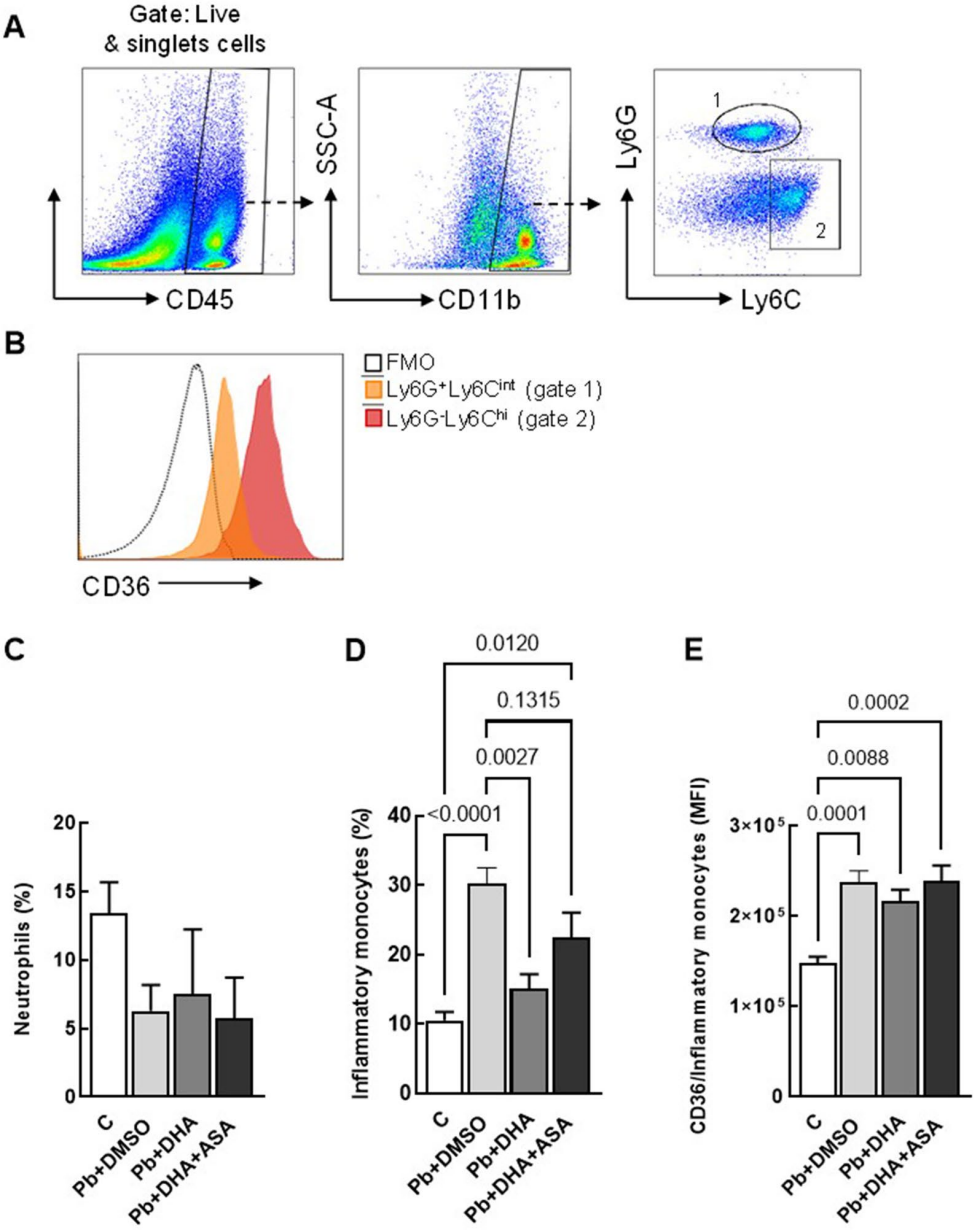
CD36 expression on monocytes in MA-ALI. Representative plots illustrating the gating strategy employed for the lung cells analysis. Following the gating on live and singlets cells, CD45^+^CD11b^+^ cell subpopulations were identified based on Ly6G and Ly6C expression in neutrophils (Ly6G^+^Ly6C^int^CD11b^+^ cells) and inflammatory monocytes (Ly6G^−^ Ly6C^hi^CD11b^+^ cells) (**A**). Histogram plot displaying CD36 expression on inflammatory monocytes (red filled) (**B**). Percentage of neutrophils (**C**) and inflammatory monocytes (**D**) in the lungs of control and *Pb*NK65 infected mice treated with vehicle (DMSO), DHA or DHA + ASA. CD36 MFI on inflammatory monocytes (**E**). Data are represented as means ± SEM of 4–7 animals in each group

Prior research has suggested that ASA can enhance CD36 expression in human macrophages [17]. In the *P. berghei* ANKA malaria model, CD36 expression has been linked to lung parasite sequestration, resulting in acute lung injury and heightened mortality [18]. However, the impact of *Pb*NK65 infection on lung function and CD36 expression remains unclear. Thus, the recruitment of inflammatory monocytes (Ly6G^−^Ly6C^int^/CD11b^+^ cells), as well as the expression of CD36 on inflammatory monocytes were evaluated. The results showed that both DHA and DHA + ASA treatment reduced the percentage of inflammatory monocytes compared to the Pb + DMSO, but only the DHA group reached a percentage comparable to uninfected animals. Moreover, the CD36 expression on inflammatory monocytes was higher in all infected groups (Fig. 5C, D). Also, the percentage of neutrophils remained similar between control and infected animals, indicating a monocytic profile of MA-ALI in this model (Fig. 5C). The results shown in this study suggest that a single dose of ASA may increase the recruitment of inflammatory monocytes, which exhibited higher CD36 expression than uninfected animals, as well as it promotes lung function impairment in the *Pb*NK65 model.

## Discussion

Besides efforts to reduce its incidence, malaria is still prevalent in tropical and subtropical countries. *Plasmodium vivax* infections are more common in the Amazon region and sub-Saharan Africa, leading to high morbidity and mortality. Among the complications, 20 to 30% of severe malaria cases caused by *P. falciparum* or *P. vivax* led to lung damage. Respiratory distress was reported in 6.7 to 30% of children [30–32], and up to 4% pregnant women [33] with falciparum malaria even after taking anti-malarial drugs. The mechanisms involved in MA-ALI have not been fully elucidated, and the use of experimental models that mimic the disease is essential to identify new therapeutic strategies and prevent complications. Although *Pb*ANKA can reproduce some features of human lung pathology, mice rapidly succumb to hyperparasitaemia or/and cerebral malaria (CM), which limits pulmonary analysis [34]. *Pb*ANKA-infected DBA/2 mice can also develop MA-ALI. Nevertheless, about 40% of deaths are associated with hyperparasitaemia [35]. The experimental model using C57BL/6 mice infected with *Pb*NK65 was able to reproduce lung oedema, hypoalbuminaemia, haemorrhage, and leukocyte infiltration seen in MA-ALI without CM symptoms [23].

Several studies have investigated the therapeutic effects of DHA in experimental malaria models. Saline-solubilized DHA treatment did not prevent death on *Pb*NK65 infection, and DHA delivery vehicle protocols were conflicting [25, 26, 36, 37]. Thus, it was further investigated whether other forms of solubilization would improve the efficacy of DHA treatment. Interestingly, the results of this study revealed that DHA was able to increase the survival rate and delay the course of parasitaemia differently, depending on its solubilization. Indeed, the instability of DHA in aqueous solution due to its complex structure has been previously described [38, 39] and might be related to higher mortality and parasitaemia observed in the DHA(Sal) group. This may be attributed to the chemical characteristics of the cosolvent DMSO, an amphipathic molecule that contains a polar domain and two apolar groups and is able to dissolve both polar and hydrophobic compounds [40]. When DHA was given in *Pb*ANKA-infected animals, both solubilized in saline or tween 80, the peripheral parasitaemia was not abrogated [26, 41]. In addition to the form of solubilization, this result can be attributed to the use of monotherapy instead of the combined treatment of DHA with piperaquine.

Although *Pb*NK65 strains have successfully induced MA-ALI, the morpho-functional impairment in this model is still poorly investigated. Herein, it was described that respiratory distress initiated on the 8-dpi due to worsening viscoelastic pressure, followed by higher static lung elastance and resistive pressure on day 9. This study focused on assessing the impact of ASA and DHA treatment on lung injury caused by malaria infection. So, mice were treated at 8 dpi, and the analyses were conducted from 9 to 15 dpi. Alveolar wall thickening oedema and infiltration of monocytes were observed as previously described [23]. Thus, the *Pb*NK65 model mimics the histopathological findings observed in post-mortem analyses of pulmonary malaria [23, 42]. The increase in viscoelastic pressure may be related to pulmonary oedema, alveolar septum thickening, and inflammatory infiltration in the lung tissue, as observed in *Pb*ANKA [43]. Higher resistive pressure and pulmonary static elastance may indicate tissue remodeling, as seen in other ALI etiologies [36, 44]. Another factor related to the inflammatory stimulus to the lung tissue occurs through the accumulation of haemozoin, which has a positive correlation with increased lung weight and alveolar oedema in experimental models, in addition to being a component observed in lung autopsy of children who succumbed to severe malaria in Africa [45].

Differently from other organ dysfunctions in severe malaria, the onset of respiratory symptoms commonly occurs after the decrease of parasitaemia [46], and the study of artemisinin derivatives in experimental models of MA-ALI might elucidate some mechanisms related to the course of the disease that remain unclear. In this context, artesunate is rapidly converted into DHA, a derivative of artemisinin with remarkable activity in preventing the development of circulating ring-stage parasites [47] and inhibiting cytoadherence and rosette formation in *P. falciparum* infection [48]. Dormoi and collaborators have previously demonstrated that DHA alone or combined with atorvastatin prolonged survival rate but could not decrease parasitaemia in a murine model of CM [26]. Indeed, in *Pb*NK65-induced MA-ALI, DHA abrogated mortality but could not eliminate parasitaemia.

Physiological lung dysfunction was accompanied by alteration in alveolar-capillary barrier permeability on 8 and 9 dpi and confirmed by the increase of protein concentration in BALF, due to extravasation of plasma protein to the lungs observed on day 9 post-infection, as previously observed on *Pb*ANKA [43] and *Pb*NK65 experimental models [23], as well as influx of mononuclear cells and increased levels of MCP-1 on BALF. Haemozoin uptake by monocytes increases inflammatory mediators’ transcription, including MCP-1 [49]. Although MCP-1 levels remained higher after DHA treatment, the decrease in lung morphological damage, viscoelastic pressure, and static lung elastance may be attributed to its anti-inflammatory properties, as described in CM [26].

A previous study has described that ASA reduced the expression of NF-κB p65 and levels of inflammatory cytokines, including IL-1β and TNF, and increased IκBα levels in a mice model of ALI triggered by hyperoxia [50]. Thus, considering that the activation of NF-κB induces the transcription of MCP-1 mRNA and culminates in MCP-1 release [51], the maintenance of BALF MCP-1 near control levels in Pb + DHA + ASA might be explained through this mechanism. When ASA acetylates cyclooxygenase-2, 15-epimers of lipoxins are generated. In this context, aspirin-triggered lipoxin A4 (ATL) mitigated lung damage in the LPS-induced ALI model. The authors found that ATL reduced leukocytes in the BALF, pulmonary myeloperoxidase activity, and MCP-1 levels by downregulating MAPK and NF-κB pathways [52]. On the other hand, ASA treatment use in subjects with LPS-induced lung inflammation did not lead to reduced MCP-1 levels in BALF [12]. However, it is important to highlight that MA-ARDS/ALI is characterized by a massive influx of monocytes instead of neutrophils, such as in ALI triggered by hyperoxia and LPS, and both humans or PbNK65-infected mice experience severe anaemia and thrombocytopenia, which is related to worsening of prognosis.

*Pb*NK65 infection also led to histological alterations in the liver, such as an intense presence of haemozoin, as well as hyperplasia of Kupffer cells, in addition to an increase in ALT and AST, and a reduction in alkaline phosphatase and serum albumin levels. These findings align with what was observed in other murine models, such as severe anaemia induced by *Pb*ANKA or *Plasmodium chabaudi chabaudi* strain AS*.* In these models, an increase in liver and spleen weight was observed, in addition to increased erythrophagocytosis, observed by staining Dylight 633 NHS ester, which binds to red blood cells [53], which is compatible with the presence of haemozoin observed in the *Pb*NK65 infection model. The presence of liver alterations is reported in up to 45% of cases of malaria and in 87% of patients who present with jaundice. In humans, histological changes are similar to those observed in the present study, with monocyte infiltration, hyperplasia of Kupffer cells, and accumulation of haemozoin and are usually attenuated after using ACT [54].

The influence of ASA on the MA-ALI outcome was also investigated, given the ambiguous effects described on CM [15, 16]. In this context, the influx of monocytes to lung tissue was maintained after DHA and ASA treatment up to 15 dpi. The entrapment of erythrocytes in the pulmonary microcirculation leads to endothelial activation and an increase in the inflammatory response, causing lung injury. Indeed, previous work has described that MA-ALI may occur even after ACT treatment [6]. Infection by *P. vivax* is also associated with MA-ALI, which most typically occurs 2 to 3 days after the beginning of anti-malarial treatment and, in some cases, can affect the patient even after the clearance of the parasite [9].

The results of this study also revealed that ASA treatment culminated in the worsening of alveolar oedema, alveolar septal thickening, inflammatory infiltrate, and atelectasis, which might be attributed to increased monocyte recruitment. A previous study analysed subpopulations of monocytes present in the inflammatory infiltrate after infection with *Pb*ANKA revealed that both CCR2^+^-CD11b^+^ interstitial macrophages and Ly6C^low^-CD11b^+^ monocytes increased 14 and 18 times, respectively, and that these populations originate from Ly6C^high^ bone-marrow monocytes [55]. Herein, ASA treatment increased the Ly6G^−^ Ly6C^high^-CD11b^+^ subpopulation compared to control, which was abrogated when animals were treated only with DHA. Moreover, CD36 expression increased on Ly6G^−^Ly6C^high^-CD11b^+^ inflammatory monocytes.

CD36 is an essential molecule in the pathogenesis of malaria, expressed in platelets, mature monocytes, macrophages, and erythroid precursors. Its expression in spleen monocytes allows the clearance of the parasite by this organ. However, its presence in platelets and endothelium leads to the formation of platelet-rich clusters that can cause obstruction or subocclusion of the microvasculature [56]. Interestingly, the results shown in this study suggest a higher percentage of inflammatory monocytes expressing CD36 can worsen lung injury in experimental MA-ALI. This agrees with what was previously described in humans, as phagocytosis of infected erythrocytes can occur by interacting with the phagocyte’s surface CD36 receptor. Moreover, mutations in this receptor have been reported in some African populations and are associated with malaria susceptibility [57]. Thus, since CD36 presents a dual role in the pathogenesis of malaria, its higher expression in *Pb*NK65-infected groups might indicate susceptibility to severe malaria in this experimental model.

## Conclusions

The findings demonstrate that the *Pb*NK65 model effectively studies lung morpho-functional parameters that mimic MA-ALI in humans. The study shows that while DHA anti-malarial treatment improved the survival rate in experimental MA-ALI induced by *Pb*NK65, it could not reverse lung morpho-functional damage. Therefore, the results suggest that DHA could be used as an anti-malarial treatment in *Pb*NK65 infection and provide an opportunity to explore the role of adjuvant therapies targeting both inflammatory processes and lung morpho-functional impairment. Additionally, administering a single dose of ASA can worsen diffuse alveolar damage and lung resistive pressure, decreasing the survival rate. Further studies are necessary to understand the mechanisms behind the harmful effects of ASA treatment on leukocyte recruitment and release of inflammatory mediators in MA-ALI.

## Supplementary Information

Supplementary Material 1: Fig S1. Liver changes after treatment with DHA and ASA. Representative photomicrographs of histological sections of the liver stained with hematoxylin–eosin from the control and *Pb*NK65-infected mice, treated with vehicle (DMSO), DHA or DHA + ASA at 9 dpi (A). Note that the infected groups show hyperplasia of Kupffer cells, the abundant presence of haemozoin (arrows), an increase in circulating cells in the sinusoids, and the interaction of leukocytes with the endothelium of the central vein (details). Serum levels of aspartate aminotransferase (B), alanine aminotransferase (C), albumin (D), and alkaline phosphatase (E). Data are represented as means ± SEM of 4–6 animals in each group. Bars: 100 μm.

Supplementary Material 2: Fig S2. *Pb*NK65 infection and treatment with DHA and ASA does not lead to CM nor to kidney injury. Representative images of brains (A) after Evans Blue injection to evaluate vascular permeability of brains. Quantification of Evans Blue dye (B). RBC (C), haemoglobin (D), hematocrit (E), platelets count (F), as well as sodium (G), urea (H) and creatinine (I) serum levels were measured at 9th dpi. N = 8–16 animals per group (A, B) and 4–9 animals per group (C–I). Data are represented as means ± SEM.

## Data Availability

The datasets used and analysed during the current study are available from the corresponding author upon reasonable request.

## Abbreviations

ΔP1, L: Resistive pressure
ΔP2, L: Viscoelastic pressure
ALT: Alanine aminotransferase
ARDS: Acute respiratory distress syndrome
ASA: Acetylsalicylic acid
AST: Aspartate aminotransferase
BALF: Bronchoalveolar fluid
CM: Cerebral malaria
COX-1: Cyclooxygenase-1
CQ: Chloroquine
DAD: Diffuse alveolar damage
DHA: Dihydroartemisinin
DMSO: Dimethyl sulfoxide
Est, L: Lung static elastance
KC: Keratinocyte chemoattractant
MA-ALI: Malaria-associated acute lung injury
MCP-1: Monocyte chemoattractant protein
*Pb*ANKA: *Plasmodium berghei* ANKA
*Pb*NK65: *Plasmodium berghei* NK65
PBS: Phosphate-buffered saline
PS: Polysorbate 80
RBC: Red blood cell
