# Supplementary figures and images for: Acetylsalicylic acid and dihydroartemisinin combined therapy on experimental malaria-associated acute lung injury: analysis of lung function and the inflammatory process
Source: Malar J. 2024 Sep 19;23:285. doi: 10.1186/s12936-024-05017-7 (PMC11414147; doi:10.1186/s12936-024-05017-7)

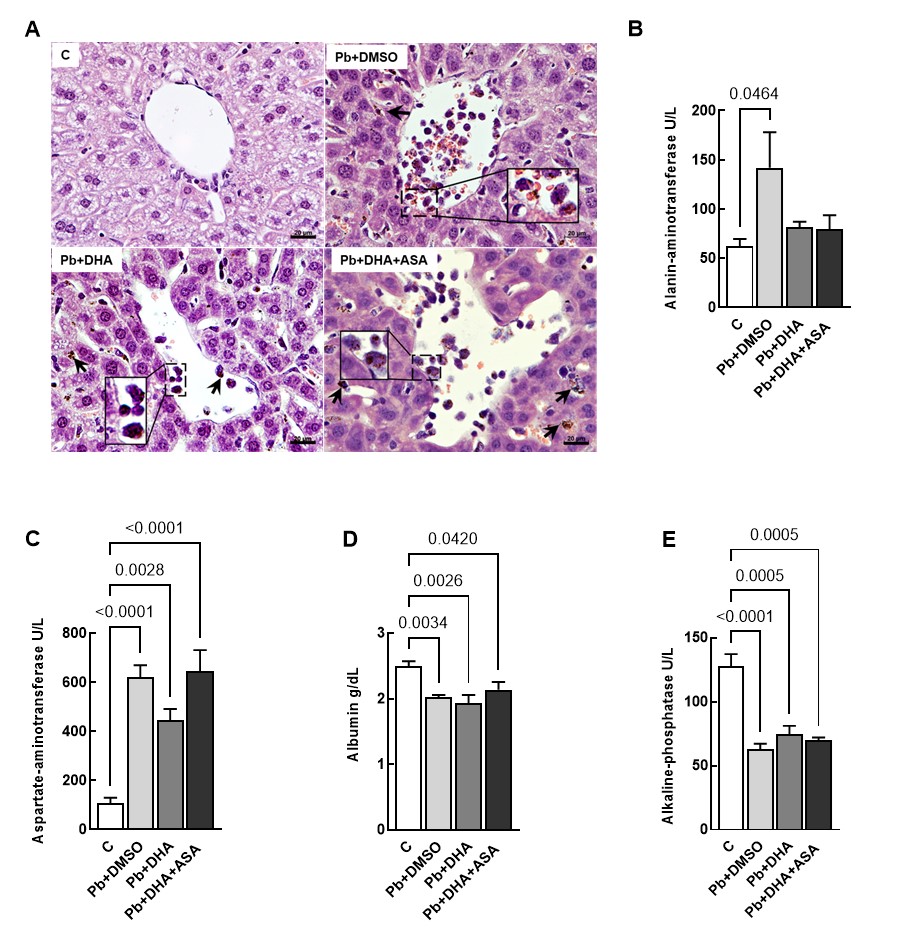

Supplement: Supplementary file 1 — Supplementary Material 1: Fig S1. Liver changes after treatment with DHA and ASA. Representative photomicrographs of histological sections of the liver stained with hematoxylin–eosin from the control and PbNK65-infected mice, treated with vehicle (DMSO), DHA or DHA + ASA at 9 dpi (A). Note that the infected groups show hyperplasia of Kupffer cells, the abundant presence of haemozoin (arrows), an increase in circulating cells in the sinusoids, and the interaction of leukocytes with the endothelium of the central vein (details). Serum levels of aspartate aminotransferase (B), alanine aminotransferase (C), albumin (D), and alkaline phosphatase (E). Data are represented as means ± SEM of 4–6 animals in each group. Bars: 100 μm. [file 12936_2024_5017_MOESM1_ESM.jpg]

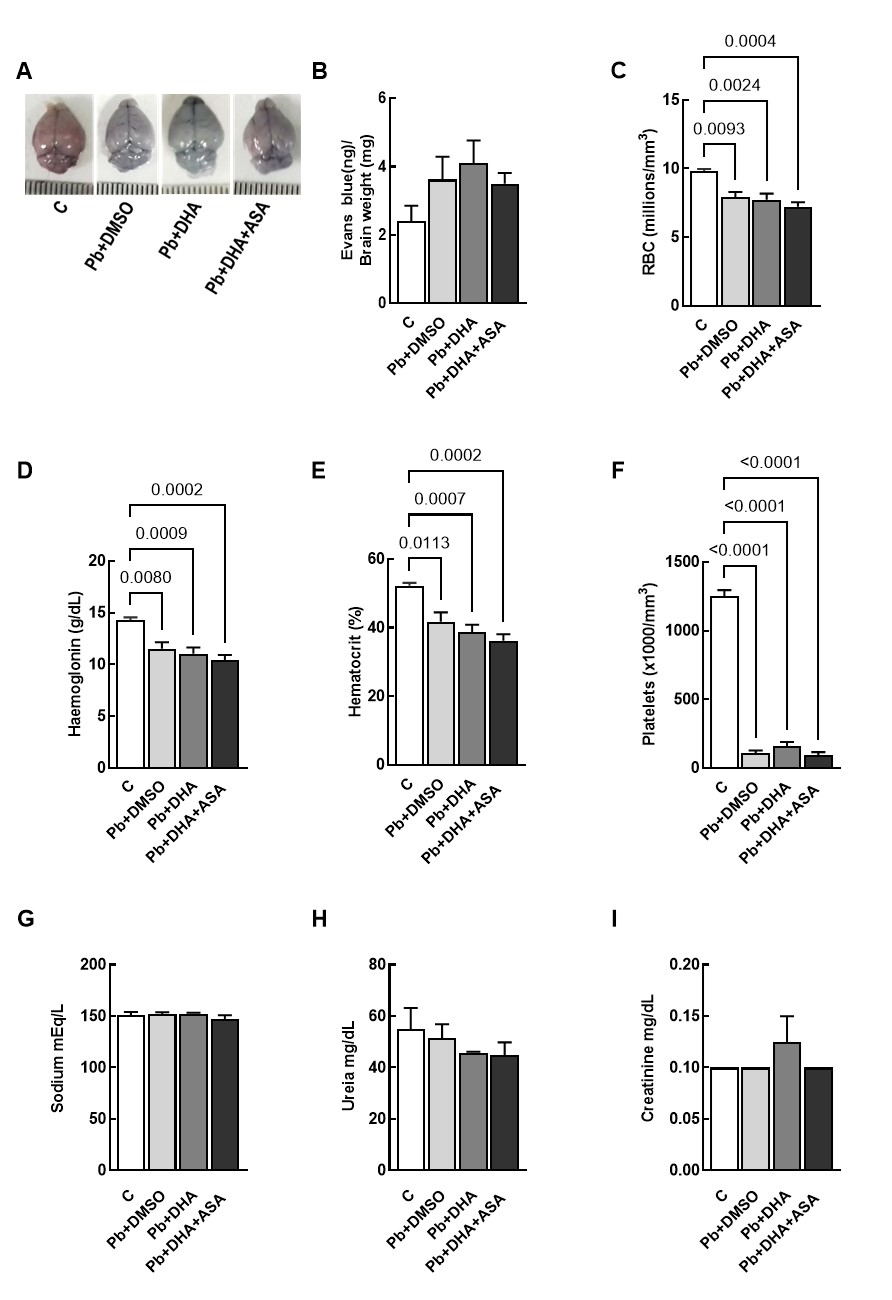

Supplement: Supplementary file 2 — Supplementary Material 2: Fig S2. PbNK65 infection and treatment with DHA and ASA does not lead to CM nor to kidney injury. Representative images of brains (A) after Evans Blue injection to evaluate vascular permeability of brains. Quantification of Evans Blue dye (B). RBC (C), haemoglobin (D), hematocrit (E), platelets count (F), as well as sodium (G), urea (H) and creatinine (I) serum levels were measured at 9th dpi. N = 8–16 animals per group (A, B) and 4–9 animals per group (C–I). Data are represented as means ± SEM. [file 12936_2024_5017_MOESM2_ESM.jpg]
